# Pivot Teeth

**Published:** 1878-07

**Authors:** F. Peabody


### PIVOT TEETH.

BY F. PEABODY.

*Read before the Kentucky State Dental Association.*

Mr. President and Gentlemen of the Association.—I was appointed to prepare a paper on surgery, but have not done so, as I understood the subject would be presented by the other member of the committee. I have, however, concluded to call your attention to a matter not down on the programme, believing that it will prove of interest to the members of the association and to the profession at large. I have always been extremely cautious about bringing anything new before the profession until I have tested by time its worth and durability, having learned by experience not only in ideas that presented themselves to me, but particularly in following those advocated by others, how often points and practices that appeared at first to be just the thing proved to my chagrin when subjected to time and use to be anything but what I hoped and expected.

The matter which I intend to bring before you, is one that has engaged my attention for the past six or seven years; during that time I find that other parties in the east have been working up the same idea, and last year Dr. Bonwill, of Philadelphia, read before the Odontological Society of New York, a paper which described the idea very clearly. As some of you may not have seen this, and as the matter will bear repeating, I propose giving you my experience from the time the matter presented itself to me up to the time when I felt it was completed, and fit to bring before the profession as an improvement in practice.

The subject on which I shall ask your indulgence, is that of grafting artificial crowns into natural roots, more generally known as pivoting teeth. I take it for granted that no one will take issue with the proposition, that where a single tooth crown is lost, if it can be replaced by an artificial crown on a strong healthy root, in such a manner that it will be satisfactory in appearance and usefulness, and free from all odor, pain and annoyance, that it is preferable to removing the root and substituting a tooth mounted on a plate; this proposition being accepted it will hold good where two or even three crowns are lost, particularly if they are in different parts of the mouth. The grafting of artificial crowns has been gradually falling into disuse. For many years the only way known of setting them, was by means of a wooden pivot, and these, when inserted with care, lasted well, particularly if the pivot had previously been immersed in creosote, a solution of chloride of zinc, or some other antiseptic fluid; they were also strengthened by having a small stiff, gold wire driven through the length of the pivot, but these wooden pivots in time absorbed the fluids of the mouth and became offensive, and the joint between the crown and root was never so tight but saliva and food found a lodgement there, and these decaying aided in decaying the root, which with the saliva soaked pivot rendered the whole anything but sweet and pleasant. From the unsatisfactory results attending the use of the wooden pivot the profession began looking around for some better method of inserting pivot teeth. Various methods were suggested and put in practice, these it is not necessary to describe as you are doubtless familiar with them, they were generally improvements on the old style, but all as far as I know are more or less objectionable, some being quite complicated and none being adapted to any but the six front teeth.

From the difficulty of saving the bicuspids by filling, the crowns of these teeth are more frequently lost than any of the teeth that show when the mouth is open. Efforts have been made to supply artificial crowns to the roots of bicuspids when the natural crowns have been crumbled away or broken off, but with such poor results (the crown generally being that intended for a cuspidati root and merely serviceable to fill the gap) that most operators prefer moving the roots and replacing the teeth on a plate, deeming it better in most respects, particularly for service. The plan which I had intended to suggest is particularly adapted to bicuspids and molars. The front teeth can be replaced in the same manner, but the labor is somewhat greater, owing to the beveled cutting edge of these teeth.

During the years 1870 and '71, when I was practicing in Rio de Janeiro, my attention was attracted to the tube teeth manufacured by C. Ash & Sons, of London. These teeth were made to be mounted on a gold plate, and each tooth has a round hole lined with platinum, which runs through its entire length. In preparing them for a plate, the teeth are ground to fit the plate, marked through each hole, a gold wire the size of the whole soldered to the marked place on the plate, the tooth slipped on to the wire, and the end of the wire riveted down on to the tooth, or the tooth fastened to the wire by sulphur or some other cement. I believe this was a primitive style of work before the days of porcelain teeth with platinum pins, but I have seen very handsome work with these teeth which is hardly excelled by the best gold work of the present day.

These tube teeth suggested a means of adapting artificial crowns to bicuspid and molar roots which I experimented with while there, and after I returned to the United States meeting with considerable success.

Taking a piece of gold wire the size of the hole in the artificial crown, and a little over a quarter of an inch in length, I bent it into the shape of the letter U; to the centre of the curved part of the U, I soldered a straight piece of wire of the same size, and a little longer than the artificial crown. We will suppose the root to be a bicuspid with two roots or canals. Having removed the root vessels and filled the canals with lead to within a little over an eighth of an inch of the gum, the remainder of each canal is enlarged to receive the U and the division between the canals cut away to a little more than the thickness of the gold wire in depth, so when the U is put in the joint where the straight piece is soldered on will be a little below the surface of the root. The U after being notched, I now plugged into the root with cohesive gold, then slipping the artificial crown on to the projecting wire, the wire was cut off almost even with the crown and the end riveted down with a small hammer—the opening in the crown having been previously countersunk.

I found in two or three of these cases that the rivet head had worn away in a few months, and the tooth had become loose on the wire, and in none of them was the joint tight between the root and the crown.

On examining one of them about a year after it had been put in, I found the wire loose in the filling. I took it out and set it in again, but this time, as the gum had come down over the edge of the root, and wept profusely, I set it in amalgam. This was about four years ago, and to-day the wire is as firm and immovable in the root as the day after it was set.

About this time a friend in Rio sent me some small pieces of carbonites or black diamonds for cutting cavities in artificial teeth for the purpose of putting in fillings. It occurred to me that perhaps I might be able to enlarge the hole in the end of these artificial crowns to receive a nut,.this with the carbonites which I had mounted as drills I found I was able to do easily. The next pivot tooth that was to be set was set with a screw and nut. A thread was cut on the lower third of the wire and a nut made to fit it. The nut was then put in the lathe (the dental engine will do as well) and turned to the size of the enlarged opening in the artificial crown, and with a few moments work ground in with emery and oil until it was a perfect fit. I procured more diamonds, and made up a number of drills varying in size from a pin to a small pea. These I find very useful not only for the purpose I have mentioned, but for enlarging the holes in old fashioned pivot crowns, and making cavities in plate and rubber teeth where it is desirable to insert gold fillings. I have also used them on blocks of teeth in rubber work where the pins have pulled out of the blocks, by converting the places that held the pins into undercut holes the same block can be replaced and held as strongly as before. In fact these diamond drills come into use in various ways, and supply a want long felt.

I am satisfied that pivot teeth set in the manner I have undertaken to describe, are better and stronger in every way than when set in any other way that has come within my knowledge. The plan has several advantages; first the amalgam in the root is easily and quickly inserted, and holds the wire more firmly than any other material with which I am acquainted. Next the wire can be placed in the root and partly filled around, then the crown slipped on and pressed to its place, the wire will in the soft state of the amalgam take any direction the crown inclines it to; and when the crown is removed the filling can go on and the wire be kept in the same direction. The crown can be tried on several times during the process of filling to see that the wire has the right direction.

Again if in filling the amalgam is left a little fuller than is required, when the tooth is finished (and this should always be the case) when the crown is slipped on and pressed firmly up to its place, particularly if it is rotated a little, the surplus amalgam will be forced between the root and the crown, taking the exact impression of the ground end of the crown, which it contains; so if the crown is not ground to fit the root perfectly, the amalgam fills up the interstices, making a joint that not even saliva can enter after the nut is put on and screwed down tightly to its place.

The amalgam should be worked as dry as possible, freed from all excess of mercury. If gold wire is used it should be covered with a thin coating of shellac varnish to prevent any danger of its being amalgamated and made brittle; platinum wire would be better than gold, as it will not amalgamate; but the best article is an alloy of platinum and iridium which is exceedingly tough.

After the pin has been set in the amalgam, and the crown pressed tightly home, (driven by a mallet if preferred) the place for the nut should be filled with oxy-chloride of zinc, and the crown held in place until it has set firmly. Whatever amalgam has been forced out from between the crown and root can then be removed and the patient dismissed until the next day; when the nut can be screwed down tight. It would not answer to put the nut on at once, as the wire might be drawn from the amalgam.

I am not in favor of the use of amalgam generally, and would be glad if something equally hard and durable could be found to take its place; but for this purpose it certainly answers better than anything else we have, and the amount that is exposed to the gums being so small, not necessarily much thicker than a sheet of paper, I am convinced it can do no harm when polished up. I have sometimes after the pin has been set, removed all the projecting amalgam, and put in its place a piece of warmed Hill's stopping or gutta percha before pushing the tooth home, but it is hardly durable enough for that purpose. I have also set the nut in Hill's stopping when it was a little to small for the countersink.

The molars can be set in the same way as the bicuspids, but for the front teeth on account of the bevelling edges the nut will not fill the countersink unless it be made very thick and cut down to the shape of the tooth, I prefer sinking it well into the crown and putting a filling over it.

These tube teeth are very natural in color and appearance the body seems to be more of a unit than porcelain teeth generally have, and finishes up well where they are ground; they have a bony appearance instead of the china look of the teeth of American manufacture. Messrs. Ash & Sons claim that they are as strong as any teeth made. I have not tried them to any great extent by fire; they have stood well where I have used them in the mouth. I procured a number of these teeth of all kinds. I countersink the holes with the diamond drill and have a number of wires prepared both for front, side and back teeth, the nuts are fitted both to the wires and the teeth, so when one is required for setting, it is but little labor to fit the pin into the root, everything else being prepared excepting the preparation of the root.

I think if the members of the profession will try this method of setting pivot teeth they will find they have something more satisfactory to themselves, and patients than they have ever had before. I submit it for what it is worth, believing on trial it will meet with your approval.

